# Rhythm May Be Key to Linking Language and Cognition in Young Infants: Evidence From Machine Learning

**DOI:** 10.3389/fpsyg.2022.894405

**Published:** 2022-05-26

**Authors:** Joseph C. Y. Lau, Alona Fyshe, Sandra R. Waxman

**Affiliations:** ^1^Department of Psychology, Northwestern University, Evanston, IL, United States; ^2^Institute for Policy Research, Northwestern University, Evanston, IL, United States; ^3^Roxelyn and Richard Pepper Department of Communication Sciences and Disorders, Northwestern University, Evanston, IL, United States; ^4^Department of Computing Science and Psychology, University of Alberta, Edmonton, AB, Canada

**Keywords:** infant cognition, language, rhythm, machine learning, non-human vocalizations

## Abstract

Rhythm is key to language acquisition. Across languages, rhythmic features highlight fundamental linguistic elements of the sound stream and structural relations among them. A sensitivity to rhythmic features, which begins *in utero*, is evident at birth. What is less clear is whether rhythm supports infants' earliest links between language and cognition. Prior evidence has documented that for infants as young as 3 and 4 months, listening to their native language (English) supports the core cognitive capacity of object categorization. This precocious link is initially part of a broader template: listening to a non-native language from the same rhythmic class as (e.g., German, but not Cantonese) and to vocalizations of non-human primates (e.g., lemur, *Eulemur macaco flavifrons*, but not birds e.g., zebra-finches, *Taeniopygia guttata*) provide English-acquiring infants the same cognitive advantage as does listening to their native language. Here, we implement a machine-learning (ML) approach to ask whether there are acoustic properties, available on the surface of these vocalizations, that permit infants' to identify which vocalizations are candidate links to cognition. We provided the model with a robust sample of vocalizations that, from the vantage point of English-acquiring 4-month-olds, either support object categorization (English, German, lemur vocalizations) or fail to do so (Cantonese, zebra-finch vocalizations). We assess (a) whether supervised ML classification models can distinguish those vocalizations that support cognition from those that do not, and (b) which class(es) of acoustic features (including rhythmic, spectral envelope, and pitch features) best support that classification. Our analysis reveals that principal components derived from rhythm-relevant acoustic features were among the most robust in supporting the classification. Classifications performed using temporal envelope components were also robust. These new findings provide *in principle* evidence that infants' earliest links between vocalizations and cognition may be subserved by their perceptual sensitivity to rhythmic and spectral elements available on the surface of these vocalizations, and that these may guide infants' identification of candidate links to cognition.

## 1. Introduction

The link between human language and cognition affords us exceptional communicative and representational power. By virtue of this link, we are able establish mental representations, ones that permit us to move beyond the present to consider the past and imagine the future, and to communicate these representations with others (Miller, 1990). Within the developmental sciences, considerable attention has been devoted to discovering how, and how early, this language-cognition link is established (Perszyk and Waxman, 2018 for a recent review). Considerable evidence has been derived from behavioral experiments measuring infant object categorization. Object categorization is a fundamental building block of cognition. In human infants, as in adults, categorization is supported by language (Gelman, 2004; Waxman and Gelman, 2009). Indeed, language supports infants' ability to form categories (Waxman and Markow, 1995; Perszyk and Waxman, 2018).

The evidence comes from a simple, yet robust object categorization task (Ferry et al., 2010, 2013; Perszyk and Waxman, 2019; Woodruff Carr et al., 2021b). During a familiarization phase, infants view a series of distinct objects, all members of the same object category (e.g., images of 8 different dinosaurs). Next, during the test phase, infants view two novel objects—one a member of the familiarized category (e.g., another dinosaur) and the other a member of a different category (e.g., a fish). The logic is straightforward: if infants detected the category-based commonalities among the familiarization objects, then they should distinguish the novel from familiar test object (as indexed by a reliable looking preference, i.e., longer looking time, for the novel object); if infants failed to detect the familiarization category, then they should fail to distinguish between the novel and familiar images. This task also allows the effect of auditory signals on object categorization to be examined: with infants viewing the same visual images in the same paradigm, the sounds paired with the familiarization images can be systematically manipulated. With this uniform design, the effect of different auditory signals on object categorization can be directly compared, even across studies by comparison of effect sizes (Woodruff Carr et al., 2021b).

Studies utilizing this task offer compelling evidence that infants' categorization is influenced by listening to language. For infants as young as 3- and 4-months, listening to their native language boosts their performance in object categorization, and does so in a way that carefully-matched acoustic signals (sine-wave tone sequences, backward speech) do not (Ferry et al., 2010, 2013). Moreover, this link to cognition is shaped by infant's own language experience. For 3- to 4-month-old infants acquiring English, listening to either English or German (a “typological cousin” to their native English with similar rhythmic properties) facilitates object categorization in the same task. In contrast, listening to Cantonese (a language typologically and rhythmically distant from English) fails to support object categorization in this task (Perszyk and Waxman, 2019). Apparently, then, infants' increasingly precise perceptual tuning to their native language (Werker and Tees, 1984; Kuhl and Rivera-Gaxiola, 2008; Peña et al., 2010; Werker, 2018) has powerful downstream consequences beyond perception alone; it also sets boundaries on which other language(s) support infant cognition.

Surprisingly, however, infants' earliest link is not restricted to language alone. Even at 4 months, as infants are narrowing the range of human languages they link to cognition, during the same object categorization task, listening to the vocalizations of non-human primates (e.g., blue-eyed black lemur, *Eulemur macaco flavifrons*) confers the same cognitive advantage as does listening to their native language (Ferry et al., 2013; Woodruff Carr et al., 2021b). Importantly, however, this link may be restricted to vocalizations of primates, our nearest evolutionary relations; It is not so broad as to include vocalization of birds (e.g., zebra-finches, *Taniopygia guttata*).

Taken together, these findings raise a compelling new question. Which acoustic features, if any, are available on the surface of human and non-human vocalizations to support very young infants in identifying which vocalizations might serve as candidate links to cognition (Ferry et al., 2013; Woodruff Carr et al., 2021b)? Focusing on the language side of this link, researchers have discovered that a strong sensitivity to rhythm, available *in utero*, is essential in the acquisition of language from the start (May et al., 2011; Langus et al., 2017; Minai et al., 2017; Gervain et al., 2021). *In utero*, the womb and other maternal tissues act as a low-pass acoustic filter, permitting lower frequency cues including rhythm and stress patterns, but not the higher-frequency cues that encode segmental detail, to be transmitted (Lecanuet and Granier-Deferre, 1993). Within hours of their birth, infants distinguish languages from the same rhythmic class as their native language, preferring them over languages with non-native rhythmical patterns (Mehler et al., 1988; Nazzi et al., 1998). Within the first year of life, rhythm continues to be instrumental (Christophe et al., 2001), enabling infants to segment the continuous speech stream into words (Johnson and Jusczyk, 2001) and to discover other structural linguistic properties including word order (Gervain and Werker, 2013) and syntactic structures (Nazzi et al., 2000). There is also strong neurophysiological evidence for the importance of speech rhythm. Neurons in the auditory cortex oscillate at frequencies that entrain speech rhythm. This entrainment, which enables infants to extract hierarchical information, including lexical stress, syllabic structure and syntactic patterns (Goswami, 2019), evident already at 4 months and develops throughout infancy (Attaheri et al., 2022), continues to support language processing in adulthood (Poeppel and Assaneo, 2020).

Thus, the power of rhythm is clear: Rhythm supports language acquisition from the start and continues to support language processing into adulthood (Gleitman and Wanner, 1982; Morgan and Demuth, 1996; Hilton and Goldwater, 2021).

Because rhythm is crucial in early language acquisition, there is reason to suspect that it may be instrumental in guiding infants to identify candidate links to cognition. There is strong evidence that speech rhythm (including that of their native language and others from the same rhythmic class), engage infant attention robustly (Jusczyk et al., 1993; Sansavini et al., 1997; Höhle et al., 2009; Räsänen et al., 2018). Finally, rhythmic properties that occur in both human language and mammalian vocalizations have been documented (Kotz et al., 2018; Ravignani et al., 2019).

This behavioral evidence of infants' sensitivity to rhythm, coupled with cross-species findings on shared rhythmic features across language and non-human vocalizations, leads to an intriguing hypothesis: that there are rhythmic properties present at the surface of languages and non-human vocalizations, that would, in principle, support 3- to 4-month-old infants in identifying them as candidate links to cognition.

Here, we provide the first test of this hypothesis. Implementing a supervised machine-learning (ML) approach, we trained a series of ML models, using acoustic features derived from a collection of audio samples of human languages and non-human vocalizations, to classify samples into classes of signals that either support infant cognition (i.e., English, German, and lemur vocalizations) or do not (i.e., Cantonese, and zebra finch vocalizations), from the vantage point of 3- to 4-month-old English-acquiring infants (Ferry et al., 2010, 2013; Perszyk and Waxman, 2016, 2019; Woodruff Carr et al., 2021b). With these models, we tested the hypothesis that rhythmic features, present at the surface of the input, support the training of the distinguishing signals that do, and do not, support cognition. As comparison, we also tested two other fundamental classes of vocal properties, namely (1) spectral envelope features and (2) pitch features. Spectral envelope features are associated with vocal configurations that differ across languages segmentally (e.g., in terms of consonant and vowel repertoire) and across species (e.g., laryngeal vs. syringeal vocalizations) (Mogran et al., 2004; Cheng et al., 2012; Andén and Mallat, 2014; Fedurek et al., 2016). Pitch features represent fundamental vocal properties across species (Belin, 2006), as well as speech intonation, another fundamental aspect of prosody central to infant language acquisition (Nooteboom, 1997).

## 2. Methods

### 2.1. Materials: Vocalization Dataset

Our modeling dataset consisted of a total of 3,197 audio samples (Table 1) of human languages and non-human vocalizations for which links to cognition (or the lack thereof) have been attested behaviorally thus far in 4-month-old infants (Ferry et al., 2010, 2013; Perszyk and Waxman, 2016, 2019; Woodruff Carr et al., 2021b).

**Table 1:** Descriptive statistics of dataset for vocalizations that do (+) and do not (−) support object categorization, from the vantage point of 4-month-old English-acquiring infants.

	**Vocalization**	**Label**	***n***	**Duration (s):**
				**Mean (SD)**
Human	English	+	703	1.23 (0.78)
	German	+	369	2.62 (1.95)
	Cantonese	−	1,634	1.94 (0.99)
Non-human	Lemur	+	122	1.55 (0.48)
	Finch	−	369	9.54 (4.59)

Language audio samples were utterance-length recordings produced by multiple female native speakers of English, German, and Cantonese, in their respective languages, using an infant directed speech (IDS) register in interactions with a young child. These audio samples were high-quality recordings from three different publicly available or private IDS corpora. Samples of American English were parts of a multilanguage corpora collected for the purpose of examining aspects of universality of IDS across cultures and societies (Hilton et al., 2022). Samples of German were from the Konstanz Prosodically Annotated InfantDirected Speech (KIDS) Corpus (Zahner et al., 2016), collected from a semi- structured mother-infant play situation where mothers were given a picture book and some other toys, which they could use according to their infant's interest. Cantonese samples were from the dataset of a study examining functions of acoustic-phonetic modifications in IDS (Wang et al., 2021). The Cantonese IDS samples were collected from a semi-structured caregiver-child interaction task, where various toys were given to the female caregiver to elicit keywords of interest, while she played with the child.

Audio samples of non-human vocalizations consisted of lemur and zebra finch vocalizations. Samples of lemur vocalizations were from a private collection of lemur vocalizations collected for a sound art project (Mercer, 2012), collected from single semi-free-range lemurs from the lemur habitat in the Duke University Lemur Center. Zebra finch vocalization samples were from a publicly available database of zebra finch songs (Laboratory of Vocal Learning at Hunter College, 2015), which have also been analyzed in prior acoustic studies (Tchernichovski et al., 2001; Isomura et al., 2019).

Descriptive statistics of our vocalization dataset are presented in Table 1.

### 2.2. Acoustic Feature Extraction

A series of multivariate acoustic features were extracted from each of the vocalization samples, to serve as input in subsequent ML classification. Before feature extraction, all audio samples were first normalized in intensity (80 dB) and resampled to a sampling rate of 22,050 Hz. Since the duration of each vocalization sample varies, the duration of each vocalization was normalized by repeating the audio samples until it reaches 9.54 s (i.e., samples), the maximum duration among all vocalization samples. Next, from each time-normalized vocalization sample, we extracted three series of acoustic features that have been shown to primarily represent *rhythmic, spectral envelope*, or *pitch information*, respectively (e.g., Hilton et al., 2022). The three series of acoustic features.

First, for *rhythmic* features: four types of acoustic features were derived from all vocalization samples to comprehensively capture aspects of rhythm, namely:

1. The speech envelope spectrum (ENV) represents temporal regularities correlating to rhythmic properties of the signal (Tilsen and Johnson, 2008; Poeppel and Assaneo, 2020; Hilton and Goldwater, 2021). For each vocalization sample, the vocalic energy amplitude envelope was first derived. To derive the envelope, the raw time series was first chunked into consecutive bins of 1 s. Following Tilsen and Arvaniti (2013), the time series of each chunk was filtered with a passband of 400–4,000 Hz to de-emphasize non-vocalic energy such as glottal energy (including the f0) and obstruent noise. The bandpass-filtered signal was then low-pass filtered with a cutoff of 10 Hz to represent the envelope. The frequency decomposition of the envelope was then computed. First, the envelope was downsampled by a factor of 100 and windowed using a Tukey window (*r* = 0.1) to aid further spectral analyses. The envelope was then normalized by subtracting the mean and rescaled to have minimum and maximum values of −1 and 1, respectively. A fast Fourier transform was first applied to the normalized envelope which was also zero-padded to a 2,048-sample window. The spectra across all 1-s chunks were then averaged to form the envelope spectrum of the vocalization sample and included as features.
2. The intrinsic mode functions (IMFs) were further computed from the time-varying speech envelope (as described above) using empirical mode decomposition (EMD), representing syllabic (IMF1) and supra-syllabic-level (IMF2) fluctuations relevant to speech rhythm (Tilsen and Arvaniti, 2013). The frequency decompositions of IMF1 and IMF2 (i.e., the averaged power spectrum density of 1–10 Hz from the frequency decomposition all IMF1s and IMF2s across all 1-s envelope bins of each vocalization sample) were included as features. We selected a bin duration of 1 s to maximally eliminate the representations of slower prosodic information (e.g., intonation) and mixtures of tempos and variations in rhythmicity not relevant to the syllabic and supra-syllabic rhythm (Tilsen and Arvaniti, 2013).
3. The temporal modulation spectrum (TMS) is the frequency decomposition of the temporal envelope of a signal that reflects how fast sound intensity fluctuates over time (Ding et al., 2017). Temporal modulation of lower frequencies (<32 Hz) is a primary acoustic correlate of perceived rhythm in speech (Greenberg et al., 2003; Goswami and Leong, 2016), which contributes to speech intelligibility (Elliott and Theunissen, 2009). For each vocalization sample, the raw time series was first chunked into consecutive bins of 1 s. The TMS of each 1-s bin was then computed using the procedure and MATLAB script from Ding et al. (2017). In the procedure, the sound signal in each bin was first decomposed into narrow frequency bands using a cochlear model and then from each band the temporal envelope was extracted. The extracted envelopes were rescaled using a logarithmic function, and were then converted into the frequency domain by the Discrete Fourier Transform (DFT). The TMS was the root-mean-square of the DFT of all narrowband power envelopes. The TMS features of each vocalization sample were taken as the average TMS of all bins.
4. The wavelet time scattering (WTS) representations are low-variance representations of time-frequency properties of sounds including amplitude and frequency modulations of acoustic signals (Andén and Mallat, 2014; Andén et al., 2015). The WTS is resistant to time-warping deformations, and is therefore advantageous to be used in machine learning since as class discriminability is not sacrificed in the transformation. The WTS has been used in machine-learning work in phoneme recognition and music genre classification (Andén and Mallat, 2014), and more recently, in the detection of speech impairments based on speech signals (Lauraitis et al., 2020). WTS representations of each vocalization sample were computed using the scatteringTransform function on MATLAB, averaged across WTS transformations on consecutive 1-s chunks of the raw time series. In the WTS transformation, the acoustic signal was decomposed by filtering the time series signal using a constant-Q wavelet filter bank. Different layers of wavelet convolution transform the signal into scattering coefficients consistent of multiple orders. The second-order scattering coefficients (WTS2), representing larger-scale acoustic structures like amplitude and frequency modulation (Andén and Mallat, 2014), were taken as features.

Second, for *spectral envelope* features: two types of acoustic features were derived from all vocalization samples to comprehensively capture acoustic properties representing vocal configurations:

1. The mel-frequency cepstral coefficients (MFCC) are cepstral representations of the audio sample that concisely describe the overall shape of a spectral envelope as perceived by human. While the MFCC has been the state-of-the-art of speech recognition, representing configurations of the vocal tract in speech, it has also been used to represent configurations of the vocal tract across other mammalian species, including primates (Fedurek et al., 2016). The MFCC is also a good representation of the syringeal properties of birds (Cheng et al., 2012). We derived the MFCC using the mfcc function of the Audio Toolbox in MATLAB, with analysis windows that spanned 50 ms and overlapped with adjacent analysis windows for 25 ms. This function first took the spectrum of the data in each analysis window using the Fourier transform, and then filtered the powers of the spectrum through a mel filter bank, linearly spaced across the first 10 triangular filters and logarithmically spaced in the remaining filters. The amplitude of the discrete cosine transform of the logged mel-transformed spectral powers were taken as the MFCC, and concatenated across all analysis windows for each vocalization sample.
2. The fist-order scattering coefficients (WTS1) were features derived from WTS representations described in the previous section; these capture the spectral envelope of sounds which are related to segmental features (i.e., consonants and vowels) (Andén and Mallat, 2014).

Third, for *pitch* features, fundamental frequency (f0) contour for each vocalization sample were derived to represent how pitch varies across the duration of the vocalization. For each vocalization sample, a raw f0 contour was first derived using the pitch function of the Audio Toolbox in MATLAB. f0 values of the contour were estimated using a Normalized Correlation Function algorithm (Atal, 1972), with analysis windows that spanned 50 ms and overlapped with adjacent analysis windows for 25 ms, and were taken as pitch features.

### 2.3. Machine Learning Classification Pipeline

A total of four sets of classification models were performed, each designed to classify vocalizations that do (+cognition) and do not (−cognition) support object categorization, from the vantage point of 4-month-old English-acquiring infants (Ferry et al., 2010, 2013; Perszyk and Waxman, 2016, 2019; Woodruff Carr et al., 2021b). We first performed classifications using all classes of features combined together in a single inclusive model (*full* model). Performance of the full model will identify whether these acoustic properties distinguish vocalizations that support infant cognition from those that do not. We then performed three more specific classifications, each using one of the three feature classes (i.e., spectral envelope, rhythmic, or pitch features). Performance of these models will identify which classes of acoustic features, if any, successfully distinguish vocalizations that support cognition from those that do not.

Since the number of vocalizations varied across types (see Table 1), a Monte Carlo cross-validation (MCCV) procedure was performed to avoid imbalanced classification. The MCCV involved an undersampling procedure which randomly selected 120 vocalization samples each from those that do (English, German, and Lemur vocalizations) and do not (Cantonese and Zebra Finch vocalizations) support cognition (i.e., a total of 240 samples). Each type of vocalizations was represented equally in the two classes (i.e., 60 Cantonese, 60 Zebra Finch vocalization, 40 English, 40 German, and 40 lemur vocalization samples). The 240 samples were then split into training and testing sets with stratified sampling in a 75:25 ratio. The MCCV also allowed us to minimize optimistic bias in the classification (Raschka, 2018) so as to objectively evaluate its performance. 100 iterations of MCCV were performed.

In each iteration of MCCV, a principal component analysis (PCA) was first performed on the input acoustic features of the particular model, so as to reduce the dimensionality of the data. PCA was performed only on the training set to avoid data leakage. Principal component (PC) scores that collectively explain 95% of total variance of the training set was selected as training features for subsequent classification, whereas acoustic features from the test set were separately transformed into PC scores using the transformation matrix of the PCA results.

Classification was then performed using an ensemble modeling approach of ML, which selected the optimal classifier for the particular MCCV sub-sample, out of a classifier array of: (1) LASSO, (2) decision tree (DT), (3) support vector machine (SVM), (4) ridge regression (Ridge), and (5) Naïve Bayes (NBC). The selection of the optimal classifier was performed using a nested four-fold cross-validation procedure, which further divided the test set into four-folds. The five classifiers were trained using three out of the four-folds of the data to classify vocalizations that do and do not support cognition, while being blind to the actual specific vocalization type (i.e., language or non-human species). The training of these classifiers was then validated on the remaining fold. The process was repeated four times until all four-folds were validated. To maximize classification performance, hyper-parameter tuning for each classifier was also performed during the same nested cross-validation procedure using a grid search approach, which repeated the training and validation using all combinations of the following hyper-parameters: (1) LASSO (λ:{0.1,1,10,100}); (2) DT (minimum leaf size, 10 intervals in the log-scaled range between 1 and 67); (3) SVM (C: {0.01,0.1,1,10}; Kernel: {linear, rbf}); (4) Ridge (λ:{0.1,1,10,100}); (5) NBC: normal, kernel NBC). The combination of classifier and hyper-parameters which achieved the highest accuracy on the validation across the four-folds were selected as optimal. The optimal classifier and hyper-parameters were then used for training on the whole training set, and were then used to predict the labels of the test set. Based on such prediction, metrics of classification performance were computed, namely (1) Area Under the Curve (AUC) of a Receiver Operating Characteristics curve, (2) prediction accuracy (ACC), (3) sensitivity, and (4) specificity. Overall performance of each model was computed by averaging the AUC, ACC, sensitivity, and specificity values of all 100 MCCV iterations.

**Figure 1:**
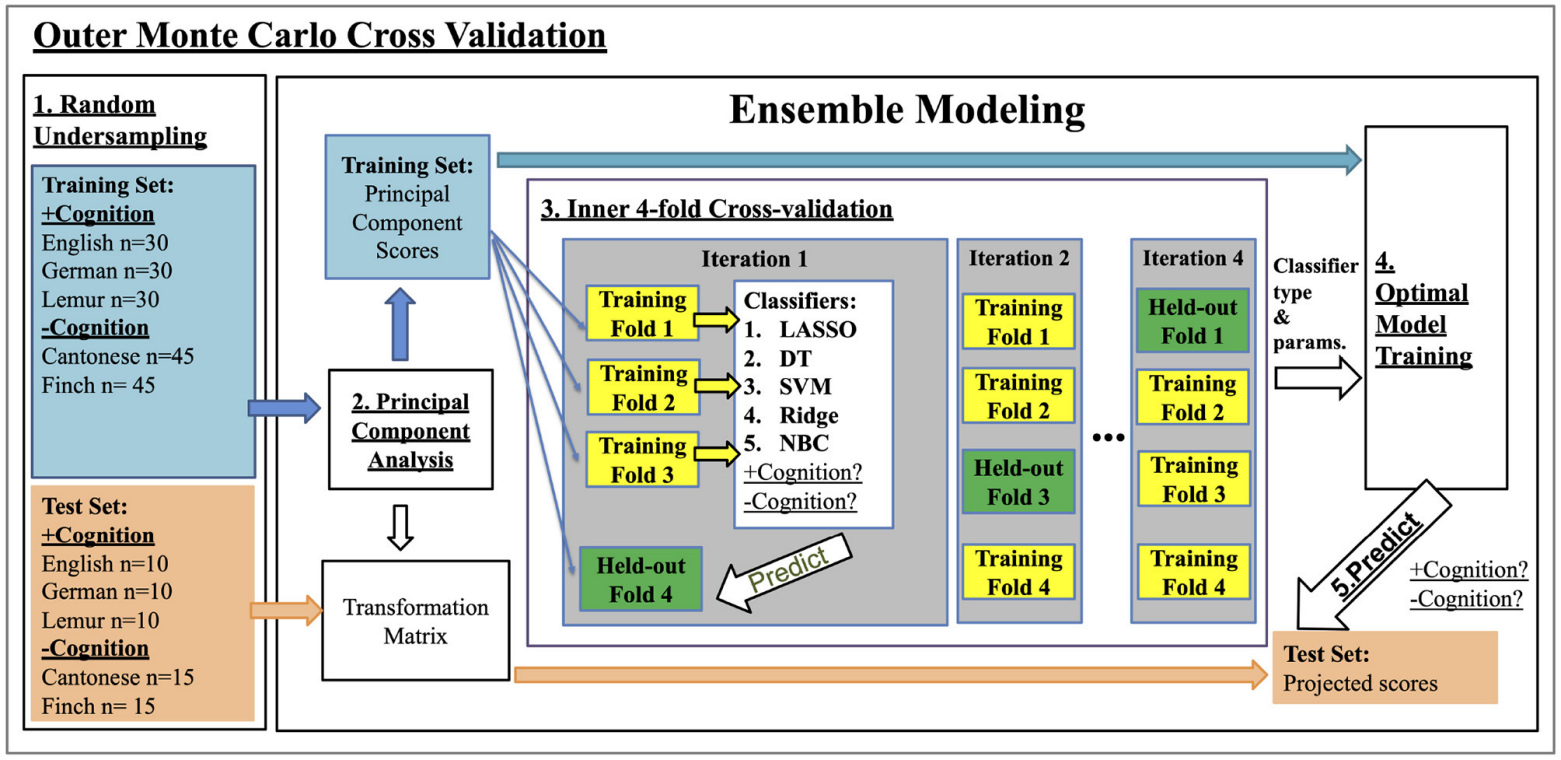
Nested Cross Validation Schema: training classifiers to classify +cognition vs. -cognition vocalizations. Step 1: In each of the 100 outer Monte Carlo Cross Validation iterations, we used an undersampling procedure to randomly select a total of 240 vocalizations from those that do (+cognition) and do not (-cognition) support cognition. Each type of vocalization was represented equally within the two classes (i.e., 60 Cantonese, 60 Zebra Finch vocalization, 40 English, 40 German, and 40 lemur vocalization samples). The 240 samples were then split into training and test sets with stratified sampling in a 75:25 ratio. Step 2: A principal component analysis (PCA) was performed on the input acoustic features (see Section 2.2) of the training set. Principal components (PCs) with scores that collectively explain 95% of total variance of the training set were selected as training features. Acoustic features from the test set were transformed into PC scores using the PCA transformation matrix calculated on the training data only. Step 3. An inner four-fold cross-validation procedure was performed to selected the optimal classifier type and parameters. We divided the training set into four-folds, and trained different combinations of classifier type and parameters using three out of the four folds of the data. The resulting models were validated on the remaining held-out fold. The process was repeated for four iterations with a different heldout fold each time. The combination of classifier type and parameters that achieved the highest accuracy in the inner cross-validation were selected as optimal. Step 4: The optimal classifier and parameters were then used for training on the whole training set. Step 5. The model from Step 4 was validated by predicting the +cognition or -cognition labels of the test set from step 1, after being transformed in step 2. The numbers calculated in Step 5 are reported in Table 2.

Schema of the MCCV and nested cross-validation procedure is visualized in Figure 1.

Performance of each model was further evaluated using a permutation approach, which involved randomizing the classification labels (+cognition vs. −cognition) while repeating the classification 1,000 times in each of the 100 MCCV sub-samples. The percentage of AUC values across all 100,000 permutations (1,000 randomizations × 100 MCCV iterations) which was equal to or higher than the actual mean AUC value was taken as the *p*-value of the model.

All machine learning procedures were performed in MATLAB, using classifier and hyperparameter tuning, and PCA functions provided by the *Statistics and Machine Learning Toolbox*.

## 3. Results

**Figure 2:**
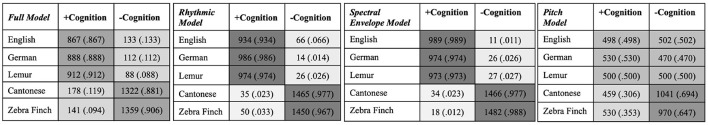
Confusion Matrices: classification of English, German, Lemur, Cantonese, and Zebra Finch vocalizations into classes of vocalizations that do (+Cognition) and do not (-Cognition) support object categorization.

Classification metrics are presented in Table 2. Figure 2 presents the confusion matrices on the percentage of each type of vocalizations being classified as those which do and do not support object categorization across the four models, from the vantage point of 4-month-old English-acquiring infants.

**Table 2:** Classification results, expressed as median area-under-the-curve (AUC) values, Sensitivity, Specificity, and Accuracy for each model.

**Model**	**AUC**	**Sensitivity**	**Specificity**	**Accuracy**
Full	0.9030^***^	0.8937	0.8890	0.8913
Rhythmic	0.9939^***^	0.9717	0.9647	0.9682
Spectral envelope	0.9955^***^	0.9827	0.9787	0.9807
Pitch	0.6287^***^	0.6703	0.5093	0.5898

***p < 0.001 in permutation test.

The *full* model performed successful classifications, achieving an AUC of 0.9030, ACC of 0.8913, sensitivity of 0.8937, and specificity of 0.8890. It also achieved statistical significance, as per the permutation test on AUC (*p* < 0.001). This is consistent with the possibility that there are acoustic properties, present at the surface among human language and non-human vocalizations, that contribute to the identification of candidate links to cognition.

The *rhythmic* model achieved robust classifications, with an AUC of 0.9939, ACC of 0.9682, sensitivity of 0.9717, and specificity of 0.9647. It was statistically significant, as per the permutation test on AUC (*p* < 0.001).

Classifications in the *spectral envelope* model were also robust, achieving an AUC of 0.9955, ACC of 0.9807, sensitivity of 0.9827, and specificity of 0.9787. Its AUC value also achieved statistical significance (*p* < 0.001).

These results may suggest that both rhythmic and spectral envelope features are among acoustic properties shared by human languages and non-human vocalizations which may be identified as candidate links to cognition.

In contrast, although the pitch model also achieved statistical significance (*p* < 0.001), its AUC of 0.6287 is indicative of “poor classification” (Hosmer et al., 2013). Its poor performance is also indicated by its near chance-level ACC (0.5898) and specificity (0.5093), although its sensitivity (0.6703) is slightly above chance. These results challenge our prediction that pitch may also play a role in identifying candidate links to cognition among human languages and non-human vocalizations.

## 4. Discussion

The current study was designed to harness the power of a supervised ML approach to address a fundamental developmental question: Which acoustic features, if any, are available on the surface of human and non-human vocalizations to support very young infants in identifying which vocalizations might serve as candidate links to cognition. Focusing on three classes of acoustic information (rhythmic, spectral envelope, and pitch), we asked (a) whether ML models could be trained to perform classifications that reliably distinguish vocalizations that support cognition from those that do not, and (b) whether rhythm or other any other class(es) of acoustic information was sufficient to support that classification.

### 4.1. Full Model

Consider first, the performance of full model. This model, which used rhythmic, spectral envelope, and pitch features combined, successfully classified vocalizations that support infant cognition from those that do not. This success held up both for human languages and non-human vocalizations. It should be noted that our models were supervised to utilize just *some* of the acoustic features, if any, that are common among vocalizations that support cognition to perform classification. Therefore, the success of the classification does not implicate that English and German resemble lemur vocalizations more than Cantonese *overall* acoustically. Instead, this successful classification, especially in the face of the considerable acoustic variability across these vocalizations, suggests there are indeed *some* common acoustic features, available on the surface of human and non-human vocalizations which support very young infants in identifying which vocalizations might serve as candidate links to cognition.

We turn next to test which class(es) of acoustic properties might best signal these candidate links.

### 4.2. Rhythmic Model

The rhythmic model, like the full model, achieved robust classification, successfully distinguishing vocalizations that do, and do not, support cognition from the vantage point of a 4-month-old English-acquiring infant (i.e., English, German, and lemur vocalizations vs. Cantonese and zebra finch vocalizations).

This outcome is consistent with robust evidence of the importance of rhythmic properties in human languages and non-human vocalizations. It also mirrors the behavioral evidence regarding infants' earliest links to cognition (Perszyk and Waxman, 2019).

Especially intriguing is that the new evidence, reported here, is consistent with proposals of parallels between rhythmic features instrumental to both human and non-human vocalizations (Ramus et al., 2000; Tincoff et al., 2005; Ravignani et al., 2019). From an acoustic perspective, non-human animals' sensitivity to rhythm is well-documented (Ravignani et al., 2019). Moreover, parallels in “babbling” of infant bats and humans suggest that rhythmic motor activity may be foundation for basic rhythmic structures across mammalian vocalizations (Knörnschild et al., 2006; Ravignani et al., 2019). In addition, human and non-human animals alike demonstrate neural entrainment to rhythm in vocalizations (Patel et al., 2009; Schachner et al., 2009). In humans, these neural oscillations are essential to identifying linguistic structure (Poeppel and Assaneo, 2020). Neural entrainment appears to be subserved by the frontostriatal brain circuitry in both humans and non-human animals, suggesting that it is not language specific (Kotz et al., 2018). This observation raises the intriguing possibility that for infants as young as 3- or 4-months of age, who cannot yet parse individual words from the ongoing sound stream, rhythm provides an entry point for identifying candidate links to cognition by establishing an early template according to infant's native rhythmic properties. Auditory signals that may conform to this native rhythmic template, such as speech from rhythmically similar foreign languages or even non-human vocalizations, may therefore be initially linked to cognition.

Indeed, we suspect that this early native rhythmic template may engage attentional mechanisms in such a way as to support infants' precocious language-cognition link. There is considerable evidence that rhythm engages infant attention (Jusczyk et al., 1993; Sansavini et al., 1997; Höhle et al., 2009; Räsänen et al., 2018) and that attention to speech rhythm is crucial to the acquisition of language (Gervain et al., 2021), highlighting distinct linguistic elements and relations among them (Soderstrom, 2007; Spinelli et al., 2017). But even more to the point, listening to their native language and to lemur vocalizations engages infants' attention neurally, as indexed by 4–9 Hz neural oscillatory activities (Woodruff Carr et al., 2021a). This rhythm-sensitive heightened attention may be a mechanism that supports infants' identification of which signals are candidate links to cognition. Additional work is required to clarify how attentional mechanisms and rhythmic properties guide infants as they discover the language-cognition link.

### 4.3. Spectral Envelope Model

The results of the ML model reported here suggest that information in the spectral envelope also yielded robust classifications. This outcome, although unanticipated, suggests that spectral envelope properties successfully classified vocalizations that support infant cognition from those that do not. This is interesting because spectral envelope features richly represent acoustic properties of speech segments (Mogran et al., 2004; Andén and Mallat, 2014) that young infants may not yet represent. Infants' sensitivity to spectral properties appears to emerge later than their sensitivity to rhythmic features (Kuhl and Rivera-Gaxiola, 2008; Werker, 2018). Thus, the current ML results may best be interpreted to suggest that spectral envelope features, whenever they do become available to infants, may be among those infants use to identify candidate links to cognition.

The success of the spectral envelope model in classifying the non-human vocalizations is not unexpected. Spectral envelope features represent vocal configurations across species (Mogran et al., 2004; Fedurek et al., 2016). For example, the physiologic distinction laryngeal (human and non-human primates) and syringeal (birds) vocalizations may be represented acoustically in spectral envelope features in the model. This raises an intriguing possibility: that infants' earliest links to cognition reflect an evolutionarily ancestral route, one that confers cognitive advantage through primate-general attentional mechanisms (Perszyk and Waxman, 2018).

The surprising success of the spectral envelope model certainly opens new avenues for investigation. For example, in future work, it will be important to assess whether lemur vocalizations have the same facilitative effect on categorization in infants acquiring languages, like Cantonese, with both segmental inventories (hence spectral envelope features) and speech rhythm that differ systematically from those of English. Meanwhile, it remains an open question whether there are other aspects of spectral envelope properties potentially common between Cantonese and lemur vocalizations, both as mammalian laryngeal vocalizations. One intriguing possibility is that lemur vocalizations do confer some cognitive advantage to Cantonese-acquiring infants, but perhaps less robustly without lemur vocalizations conforming to the rhythmic template of Cantonese. Delineating these possibilities would further shed light on the mechanistic nature of the pathway that enables infant's earliest links to cognition.

### 4.4. Pitch Model

Pitch features, which like rhythm are also related to prosody, yielded surprisingly low classification performance. This suggests that there may be few, if any, surface pitch-relevant acoustic properties that distinguish between vocalizations that do and do not support cognition, despite that pitch is one of the most prominent features of infant-direct speech (Hilton et al., 2022) also known to engage infant's attention (Sullivan and Horowitz, 1983). There are several possible accounts for this outcome. First, it may be related to the broad acoustic variability in our corpus. After all, lemur vocalizations have higher average pitch and broader pitch range than human vocalizations (Woodruff Carr et al., 2021b). Alternatively, this may reflect a limitation more particular to our corpus. We were only able to capture the f0 contour to represent speech intonation and pitch properties of vocalization in the current models. As a result, we may have failed to capture the more dynamic intonational properties of these signals. The limited amount of information represented in the f0 contour as compared to rhythmic and spectral envelope features may also have hindered classification performance from a computational perspective. Addressing this question will require additional work that incorporates a broader and more dynamic set of measures that tap into more fine-grained vocalic properties of both human and non-human vocalizations.

### 4.5. Limitations and Future Directions

The ML approach invoked here suggests that there are indeed certain acoustic properties, present in the surface of human and non-human vocalizations, that are available, *in principle*, to support infants' identification of which vocalizations link to cognition.

This outcome, important in itself, raises new questions for future work. For example, it will be important to discover whether, as infants forge their earliest links to cognition, they use the same mechanisms, or different ones, in identifying candidate human languages and non-human vocalizations. There is reason to suspect that there may be two distinct routes, one governing the links from language and another governing the candidate links from non-human vocalizations (Owren et al., 2011; Perszyk and Waxman, 2018). First, cross-species neurophysiological work has identified two neural pathways in response to human vocal communication: a subcortical pathway shared among human and non-human primates for affective vocalizations, and another cortical pathway that appears to be specific to humans for speech (Owren et al., 2011; Ackermann et al., 2014). Second, neural and behavioral evidence from 4- to 6-month-old English-acquiring infants is consistent with the possibility that there are two distinct routes (Ferry et al., 2013; Perszyk and Waxman, 2019; Woodruff Carr et al., 2021b). To examine this hypothesis, it will fruitful for future studies to apply a ML approach separately on human languages and non-human vocalizations, as well as modeling from the vantage points of infants acquiring a language other than English. Doing so will not only merely require a larger database of human and non-human vocalizations, but crucially broader empirical behavioral evidence delineating natural classes of human and non-human vocalizations that do and do not support cognition, from the vantage points of infants acquiring different varieties of languages.

While future modeling would benefit from an expansion of empirical evidence, results of the current model nevertheless shed light on future directions of empirical studies on infants' language-cognition link. Indeed, the features identified in the current models may not represent veridically the acoustic features actually utilized by infants as they evaluate candidate links to cognition. Nevertheless, future studies could target rhythm and spectral envelope features to manipulate in the stimuli in object categorization experiments (e.g., testing with low-pass filtered vocalizations or speech chimera), so as to pinpoint acoustic properties infants utilize to evaluate candidate links to cognition empirically. Further, by testing vocalizations of a larger variety of mammalian and non-human primate species, future studies could also shed light on the extent to which the link governing non-human vocalizations and cognition in young infants is modulated by the etiological distance between the animal and humans, so as to examine the hypothesis that the link governing non-human vocalizations and cognition is an ancestral pathway that reflects the residue of evolution (Perszyk and Waxman, 2018).

## 5. Conclusion

The current results offer support for the proposal that rhythmic and spectral envelope features, available in the input of human language and of non-human linguistic vocalizations, may guide infants in identifying which signals are candidate links to cognition. This in principle evidence, important in itself, is also consistent with the possibility that infants' earliest links to cognition may be subserved by their sensitivity to rhythmic and spectral envelope properties of sounds.

## Data Availability Statement

The original contributions presented in the study are included in the article/supplementary material, further inquiries can be directed to the corresponding author/s.

## Author Contributions

JL and SW conceptualized the study. JL and AF design machine learning model. JL implemented the model. All authors interpreted the data, wrote the paper, and approved it for publication.

## Funding

This work was supported by National Institute of Health (NIH) Grant R01HD083310 to SW.

## Conflict of Interest

The authors declare that the research was conducted in the absence of any commercial or financial relationships that could be construed as a potential conflict of interest.

## Publisher's Note

All claims expressed in this article are solely those of the authors and do not necessarily represent those of their affiliated organizations, or those of the publisher, the editors and the reviewers. Any product that may be evaluated in this article, or claim that may be made by its manufacturer, is not guaranteed or endorsed by the publisher.
